# Multiplexed highly-accurate DNA sequencing of closely-related HIV-1 variants using continuous long reads from single molecule, real-time sequencing

**DOI:** 10.1093/nar/gkv630

**Published:** 2015-06-22

**Authors:** Dario A. Dilernia, Jung-Ting Chien, Daniela C. Monaco, Michael P.S. Brown, Zachary Ende, Martin J. Deymier, Ling Yue, Ellen E. Paxinos, Susan Allen, Alfredo Tirado-Ramos, Eric Hunter

**Affiliations:** 1 Emory Vaccine Center, Emory University, Atlanta, GA, 30329, USA; 2 Pacific Biosciences Inc., Menlo Park, CA, 94025, USA; 3 Pathology and Laboratory Medicine, Emory University, Atlanta, 30322, GA; 4 University of Texas Health Science Center, San Antonio, TX, 78229, USA

## Abstract

Single Molecule, Real-Time (SMRT^®^) Sequencing (Pacific Biosciences, Menlo Park, CA, USA) provides the longest continuous DNA sequencing reads currently available. However, the relatively high error rate in the raw read data requires novel analysis methods to deconvolute sequences derived from complex samples. Here, we present a workflow of novel computer algorithms able to reconstruct viral variant genomes present in mixtures with an accuracy of >QV50. This approach relies exclusively on Continuous Long Reads (CLR), which are the raw reads generated during SMRT Sequencing. We successfully implement this workflow for simultaneous sequencing of mixtures containing up to forty different >9 kb HIV-1 full genomes. This was achieved using a single SMRT Cell for each mixture and desktop computing power. This novel approach opens the possibility of solving complex sequencing tasks that currently lack a solution.

## INTRODUCTION

Human Immunodeficiency Virus Type-1 (HIV-1) is one of the most rapidly evolving pathogens (1). Although in the majority of transmission events only a single variant gets transmitted to the new host (2–5), due to the high mutation rate that HIV-1 exhibits during its replication cycles (6) and immune selection, a diverse intra-host population of variants (quasispecies (7)) rapidly evolves.

The underlying variation that establishes the viral quasispecies is crucial for adaption to selective pressures (8). Therefore, a better understanding of the dynamics of the genomic variants in quasispecies can have important implications for pathogenesis (8–12), treatment (13–17) and vaccine development (18–20). To date, HIV-1 quasispecies have primarily been studied by cloning (4,21–26) or deep sequencing (19,27–29). While the former limits the number of variants that can be analyzed, the latter limits the length of the genomic segment analyzed (because of the short length of sequencing reads) or reduces the analysis to the study of the frequency of individual polymorphisms within the population without being able to confidently deconvolute the genomic DNA sequence of each of the multiple closely-related variants that comprise it. Therefore, at the present there is a limited knowledge of the actual dynamics of an HIV-1 quasispecies based on full-genomic sequences. A solution to this would be to implement the Next Generation Sequencing (NGS) technology developed by Pacific Biosciences (PacBio^®^) called Single Molecule, Real-Time (SMRT^®^) DNA sequencing. This technology combines the deep massive parallel sequencing of NGS with long sequencing reads (currently >10 kb). However, although the long reads allow creation of finished, gapless, high quality (>QV50) genome assemblies, it is only applicable when sequences are all derived from a single genome because the relatively high error rate of base calls during the sequencing process precludes the efficient phasing of genomic sequences of multiple closely related variants.

To examine heterogeneous populations using SMRT sequencing, one can build consensus sequences from multiple passes across the same molecule. Also known as circular consensus sequencing (CCS) (30), these CCS reads exhibit a significantly lower error rate since they are derived from multiple passes of the polymerase over the same circularized DNA molecule, thereby resulting in near-final, higher-quality sequence reads. However, this approach requires continuous long reads up to 10 times the length of the region of interest. In the case of near full-length HIV-1 genomic sequences, for example, this approach would require CLR sequences 100 kb in length. A recent attempt to circumvent this problem involved PCR amplifying a series of overlapping fragments across the HIV-1 genome, such that high quality CCS reads were derived and then assembled, but the level of genetic diversity in the mixture compromises any assembly approach, particularly when closely related variants are present (31). An alternative is to derive algorithms that allow the use of the raw, single-pass CLR data to infer possible haplotypes. The higher single-pass sequence read error rate of this approach requires new analysis tools.

In the present study, an analytical algorithm was developed that allows the accurate simultaneous sequencing of at least 40 distinct full-length HIV-1 genomes on a single SMRT^®^ Cell. This is achieved by exclusive use of single-pass CLR data. This statistical approach is not limited to HIV-1 but can be applied broadly to resolving other complex sequencing problems.

## MATERIALS AND METHODS

### Samples analyzed

Samples were obtained from the participants of the Zambia Emory HIV Research Project (ZEHRP) discordant couples cohort in Lusaka, Zambia, enrolled in studies for which the associated human subjects protocols have been approved by both the University of Zambia Research Ethics Committee and the Emory University Institutional Review Board. Prior to enrollment, individuals received counseling and signed a written informed consent form agreeing to participate. The subjects selected from the cohort were initially HIV-1 serodiscordant partners in cohabiting heterosexual couples with subsequent intra-couple (epidemiologically linked) HIV-1 transmission (32–34). Epidemiological linkage was defined by phylogenetic analyses of HIV-1 gp41 sequences from both partners (35). Zambian linked recipients were identified to have a median (interquartile range) estimated time since infection (ETI) of 46 (42–60.5) days, at which time plasma samples were obtained from both the transmitting source partner (donor) and the linked seroconverting partner (recipient). All of the transmission pairs included in this study are infected with subtype C HIV-1.

### Single genome amplification

Viral RNA extraction and near full-length genome Single Genome Amplicons (SGAs) were obtained by limiting dilution RT-PCR as described previously (36,37). Viral RNA was extracted from 140 μl of plasma using the QIAamp Viral RNA mini kit (Qiagen, Limburg, Netherlands) and was used for cDNA synthesis carried out with Superscript III (Life Technologies, Carlsbad, CA, USA) and an anchored Oligo(dT)18 primer. The cDNA was used immediately for PCR amplification using the Q5 Hot Start High-Fidelity DNA Polymerase (NEB, Ipswich, MA, USA). Near full-length single genome PCR amplification was performed by serially diluting cDNA, followed by two rounds of PCR amplification, so that ∼30% of wells became positive. Both rounds of PCR were performed in 1x Q5 Reaction Buffer, 1x Q5 High GC Enhancer, 0.35 mM of each dNTP, 0.5 μM of primers and 0.02 U/μl of polymerase in a total reaction volume of 25 μl. First round primers were, 1U5Cc and 1.3’3’PlCb, and second round primers were 2U5Cd and 2.3’3’plCb (38). Cycling conditions for both reactions are 98**°**C for 30 s, followed by 30 cycles of 98**°**C for 10 s, 72**°**C for 7.5 min, with a final extension at 72**°**C for 10 min. PCR reactions were visualized by electrophoresis through 1% agarose lithium acetate at 300 V for 25 min.

### SGA mixtures for library preparation

Five SMRTbell™ libraries containing multiple HIV-1 full-length genome amplicons were constructed by pooling multiple SGAs from five different patients as follows: In library #1, 18 SGAs obtained from the chronically HIV-positive transmitting partner (donor) Z4473F were mixed together with one SGA representing the transmitted/founder (T/F) virus from the acutely infected partner (recipient) Z4473M. Similarly, in library #2, 20 SGAs from the donor, Z4248F, were mixed together with the T/F virus SGA from the recipient Z4248M. Library #3, contained a mixture of all 40 SGAs used for the first two libraries. Library #4 contained a mixture of 18 independent SGA amplicons obtained from an acutely infected HIV-1 individual (Z3576F). Finally, Library #5 contained a single full-length SGA from an acutely infected patient (R880F). The sequences of all of the genomes present in the libraries were initially obtained using Sanger sequencing (GenBank KR820394-820413, KR820417, KR820422-820440).

### Library preparation protocol

PCR products from each SGA were purified separately using the Wizard^®^ SV Gel and PCR Clean-Up System (Promega, Madison, WI, USA) and DNA was quantified using the NanoDrop^®^ ND-1000 UV-Vis Spectrophotometer (Thermo Fisher Scientific, Waltham, MA, USA). Equal amounts of DNA from each of the SGAs to be included in a library were pooled together to a final concentration of 70 ng/μl. SMRTbell libraries were generated for each pool according to protocols from the DNA Template Prep Kit 2.0 (Pacific Biosciences Inc., CA, USA cat 100-259-100). Specifically, initial repair of the amplicons was done by combining 42 μl (3000 ng) of the pooled DNA sample with 5 μl of DDR Buffer (10×), 0.5 μl of NAD+ (100×), 0.5μl of dNTP (10 mM) and 2 μl of DNA Damage Repair Enzyme (25×), and incubated at 37°C for 20 min and then at 4°C for 1 min. Then the mixture is subjected to a round of DNA purification using AMPure PB magnetic beads (Pacific Biosciences, Inc.) and eluted in 30 μl of Elution Buffer. The mixture is then subjected to End Repair reaction by adding 5 μl of Template Prep Buffer (10×), 5 μl of ATP Hi (10 mM), 2 μl of dNTP (10 mM), 5.5 μl of water and 2.5 μl of End Repair Mix Enzyme (20×). The mix is incubated at 25°C for 15min and then at 4°C for 1–2 min. Another round of DNA purification using AMPure PB magnetic beads is performed and DNA is eluted in 30 μl of Elution Buffer. The mixture is then subjected to Ligation with SmartBell Adaptors by adding 1 μl of blunt adaptors, 4 μl of Template Prep Buffer (10×), 2 μl of ATL Low (1 mM), 2 μl of water and 1 μl of T4 Ligase (30 U/μl). The mix is incubated overnight at room temperature and then heated for 10 min at 65°C to inactivate the ligase. 0.75 μl of Exonuclease III and 0.75 μl of Exonuclease VII are added to the ligation mix and incubated at 37°C for 1 h in order to remove any unligated DNA. Finally, three rounds of DNA purification using the AMPure PB magnetic beads are performed, eluting in 100 μl of Elution Buffer after first and second round, and eluting in 15 μl of Elution Buffer after the final round of purification. The quality of the library was assessed by running the sample in the Agilent 2100 Bioanalyzer system (Agilent Technologies, Santa Clara, CA, USA). Final concentrations of each library were: (i) 30.94 ng/μl with a peak at 9199 bp, (ii) 28.46 ng/μl at 9359 bp, (iii) 32.04 ng/μl at 9029 bp and (iv) 25.06 ng/μl at 9130 bp. Primer annealing and P4 polymerase binding to the SMRTbell libraries were performed. SMRT sequencing was performed on the PacBio RSII, using 2-h movies.

### Genetic variants reconstruction

- Generation of initial data set
  A *fasta* file containing the initial set of reads to be analyzed is generated with the bash5tools.py code (SMRT Analysis v2.2.0 for Ubuntu 10.04), which uses information generated by the PacBio instrument contained in the *bas.h5* and *bax.h5* files. Only reads longer than 2 kb are retained in this step.
- Alignment
  A central task to sequencing mixtures of genomes is determining consensus or estimating the single most-likely genome given a set of reads that only have sequencing errors. With a robust consensus procedure in place, the problem of sequencing mixtures might be broken into two tasks: (a) stratifying reads that are likely to have originated from different genomes and (b) estimating consensus within each genome strata to remove sequencing error. We rely on the Quiver algorithm to estimate consensus and remove sequencing error. Quiver is a process that for a candidate genome and a set of reads computes the probability of the reads given the genome using a computationally efficient algorithm that explores all possible alignments of the reads to the genome. The single best consensus genome is one that maximizes this probability. With the consensus genome estimated by Quiver, a simplified multiple sequence alignment view is generated by pairwise aligning each of the reads to the single Quiver consensus and combining the pairwise alignments into a single multiple alignment.
- *Alignment* *correction* *algorithm*
  The majority of errors in PacBio raw reads are insertions and deletions (INDELs) and so the initial phase of sequence derivation is to minimize the alignment artifacts derived from such errors. Every read in the alignment is considered a row vector in which elements are aligned to the Quiver reference and can be either ‘main positions *P’*, which are positions classified as true, or ‘INDEL positions *p’*, which are positions classified as false. If *y*, *x* and *z* are ‘main positions’, any given read of length *n* can be defined as:
  \begin{equation*} \nu = [P_1 , \ldots ,P_y ,p_{y + 1} , \ldots ,p_{x - 1} ,P_x ,p_{x + 1} , \ldots ,p_{z - 1} ,P_z , \ldots ,P_n ] \end{equation*}
  where any position in between them is an INDEL position (either a gap or a nucleotide classified by the alignment method as a sequencing error). During the alignment correction algorithm, if A is the group of ‘main positions’ (*P_i_*) and *y* ∈ *Ax* ∈*Az* ∈ *A* and *y* is the next downstream main position to *x* and *z* the next upstream position to *x*, if for any *i*, where *y* < *i* < *x* or *x* < *i* < *z*, *P_i_* in {A, C, G, T}, then the nucleotide at *P_x_* is replaced by a gap, which is considered a non-informative state in our model.
  After implementing the correction to every main position in every read, all the INDEL positions in the alignment are removed leading to corrected reads defined as:
  \begin{equation*} \nu _{{\rm corrected}} = [P_1 , \ldots ,P_y ,P_x ,P_z , \ldots ,P_n ] \end{equation*}
- Identification of positions with evidence of true diversity
  For every non-consensus nucleotide *nt* at every position *z* in the alignment, the probability for that nucleotide to be a sequencing error was defined as the complement probability to the binomial cumulative distribution with a 5% uniform rate (expected frequency for noise) where *x_nt_* is the number of observations of nucleotide *nt* (A, C, G or T) and *n_z_* is the total number of observations:
  \begin{equation*} Pb = 1 - F\left( {x_{nt} |n_z ,0.05} \right) = 1 - \sum\limits_{i = 0}^{x_{nt} } {\left( {\begin{array}{*{20}c} {n_z } \\ i \\ \end{array}} \right)0.05^i 0.95^{\left( {n_z - i} \right)} } \end{equation*}
  where *x_nt_* is the number of reads in which the specific nucleotide type *nt* (A, C, G or T) was present as a potential erroneous insertion, *n_z_* is the total number of reads for the position *z*.
  Correction for multiple comparisons is performed by estimating the positive false discovery rate (pFDR) from the *p*-values using the procedure described by Storey (14) as implemented in MATLAB R2012a (*mafdr* algorithm). Only non-consensus nucleotides with a q-value less than 0.2 were considered likely to be true polymorphisms.
- Classification of reads
  Classification of reads was performed by implementing a hierarchical clustering method. *Edit distances* between reads were determined based on the positions selected in the previous step and using only overlapping positions between reads, disregarding positions where either read has a gap. The final distance is defined as the percentage of differences over the total positions included in the calculation. Then the set of reads are linked to each other on a cluster analysis based on the calculated distances and the distance between clusters is measured following the furthest neighbor method (*linkage* algorithm, MATLAB R2012a).
  If $x \in A,\;y \in B,\;d(x,y) =$ distance between objects *x* and *y*, then the distance between *A* and *B* is $dist\left( {A,B} \right) = {\rm max}\_\left\{ {{\rm x}\in A,{\rm y}{\in} B} \right\}d\left( {x,y} \right)$.
  Based on distance between clusters, reads are classified in two subgroups separated by the largest distance (*cluster* algorithm, MATLAB R2012a).
- Recurrent analysis of subgroups
  In order to derive all of the unique sequences in the mixture steps i-v are repeated until there are no positions with significant diversity remaining within the subgroups.
- Error correction algorithms
  Although each subgroup is homogeneous, some errors in the sequence remain primarily due to bases missed during sequencing. To correct these errors, we utilize two error correction algorithms.

#### Error correction algorithm #1

This algorithm reanalyzes all of the nucleotides initially classified as potentially erroneous insertions in the raw read alignment for evidence of specific nucleotides present at frequencies significantly higher than that expected for noise considering statistically significant any nucleotide having a *q*-value lower than 0.01. Any nucleotides found to be significantly prevalent are considered real nucleotides missed by the alignment process due to low frequency in the sequencing output data and are consequently inserted in the final consensus sequence. In addition, and because these misclassified nucleotides were actually present at low frequency among reads, we performed a bootstrap analysis in which the statistical analysis was implemented and repeated over 50 subsamples containing 75% of the reads randomly selected from the alignment. After the analysis, nucleotides that exhibited *p*-values lower than 0.05 and *q*-values lower than 0.01 in at least 40 of the 50 replicates are considered true nucleotides and are incorporated into the final consensus sequence.

The algorithm proceeds as follows:

- Perform a sampling without replacement of 75% of the reads in the alignment.
- For every position *P_z_* in the initial raw read alignment, determine the frequency of each nucleotide type (A, C, G and T) that was initially classified as a potentially erroneous insertion located between positions *P_z_* and *P_z+1_*,
- Calculate the probability *Pb* for each nucleotide type (A, C, G and T) to be an error as the complement probability to the binomial cumulative distribution function as follows:
  \begin{equation*} Pb = 1 - y_{nt} = 1 - F\left( {x_{nt} {\rm |}n_z ,p} \right) = 1 - \sum\limits_{i = 0}^{x_{nt} } {\left( {\begin{array}{*{20}c} {n_z } \\ i \\ \end{array}} \right)p^i q^{\left( {n_z - i} \right)} } \end{equation*}
  where *p* is the expected frequency for noise defined as the percentile 95 from the distribution of all the frequencies determined in the previous step and *q* = 1 – *p*
- Obtain the *q*-values derived from correcting the *p*-value for multiple comparisons using the Benjamini–Hochberg FDR method (39), and select as potential true nucleotides those exhibiting a *q*-value lower than 0.01.
- Repeat steps (i) through (iii) 50 times and define true nucleotides as those found to have a *q*-value lower than 0.01 in at least 40 of the 50 replicates.

#### Error correction algorithm #2

This algorithm explores every single gap in the alignment obtained after correction with algorithm 1, and defines as a ‘real’ nucleotide any nucleotide type initially classified as potentially erroneous insertions in the raw read alignment that, while exhibiting a significant *q*-value in at least one of the 50 replicates of algorithm 1, would fill a single gap in the sequence.

The algorithm proceeds as follows:

- In the corrected alignment obtained from algorithm 1, determine the frequency of gaps at each position *P_z_* of the alignment.
- For every gap in every sequence of the alignment located in an alignment position *P_z_*, determine in how many replicates (if any) of algorithm 1 a nucleotide located between positions *P*_*z*-1_ and *P*_*z*+1_ of that sequence was found to exhibit significant *q*-value.
- Define as real nucleotides those found to have a significant *q*-value in at least 1 of the 50 replicates of algorithm 1 and located between positions *P*_*z*-1_ and *P*_*z*+1_, if and only if the frequency of gaps in position *P_z_* was lower than 5%, as calculated in step (i).

The total runtime for the analysis of a dataset of 3000 reads is ∼2.3 h with peak memory requirements of 1.1 Mb.

### Validations of the method

Validation of the analytical algorithm was performed by comparison of the DNA sequences generated by our algorithm with the DNA sequences obtained by Sanger sequencing. Alignments were built using HIV Align Tool (40) [available at http://www.hiv.lanl.gov/content/sequence/VIRALIGN/viralign.html], and then hand-edited in Geneious 6.1.4. The number of mismatches between sequences was counted and analyzed using algorithms built with MATLAB 2012a.

## RESULTS

### Overview of the sequencing run and analytical approach

The goal of the present study was to develop an analytical approach able to accurately reconstruct multiple HIV-1 genomes sequenced in parallel using only Continuous Long Reads (CLR).

**Figure 1.**
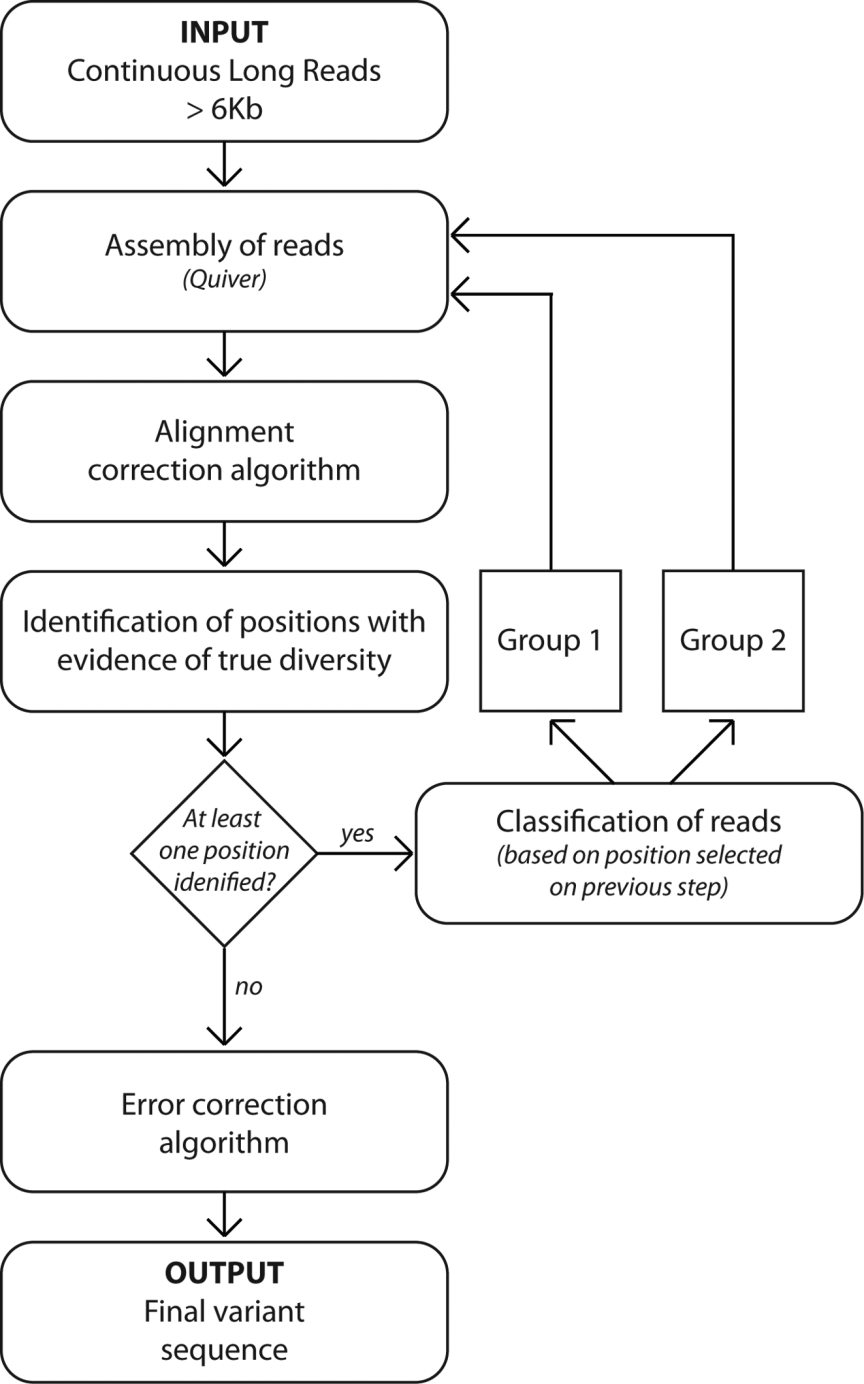
Schematic view of the main steps involved in the workflow. The workflow consists of a number of steps that initially align the Continuous Long Reads, then minimizes the impact of insertions by performing an Alignment Correction, then identifies positions with true diversity disregarding deletions, and classifies reads into two groups using a distance method based on positions selected in the previous step. The recurrent implementation of these procedures to each subgroup generated increases the homogeneity of the sequences until all the sequences contained in one subgroup were highly likely obtained from sequencing the DNA molecules with the same DNA sequence. Finally, an Error Correction algorithm is implemented over the derived sequences in order to increase their QV.

Overall, as shown in Figure 1 the analytical approach that we present here includes (i) alignment of raw CLR reads longer than 6 kb to a Quiver derived consensus, (ii) alignment correction to minimize the impact of erroneous insertions, (iii) probabilistic analysis of the diversity of each nucleotide base (A, C, G, T) at each position to minimize the impact of erroneous deletions, (iv) classification analysis using differential weighting of each position based on the preceding diversity analysis, which minimizes the impact derived from erroneous substitutions and (v) an error-correction algorithm that focuses on deletions in order to correct residual errors and achieve high levels of accuracy.

### Assembly of reads and alignment correction

**Figure 2.**
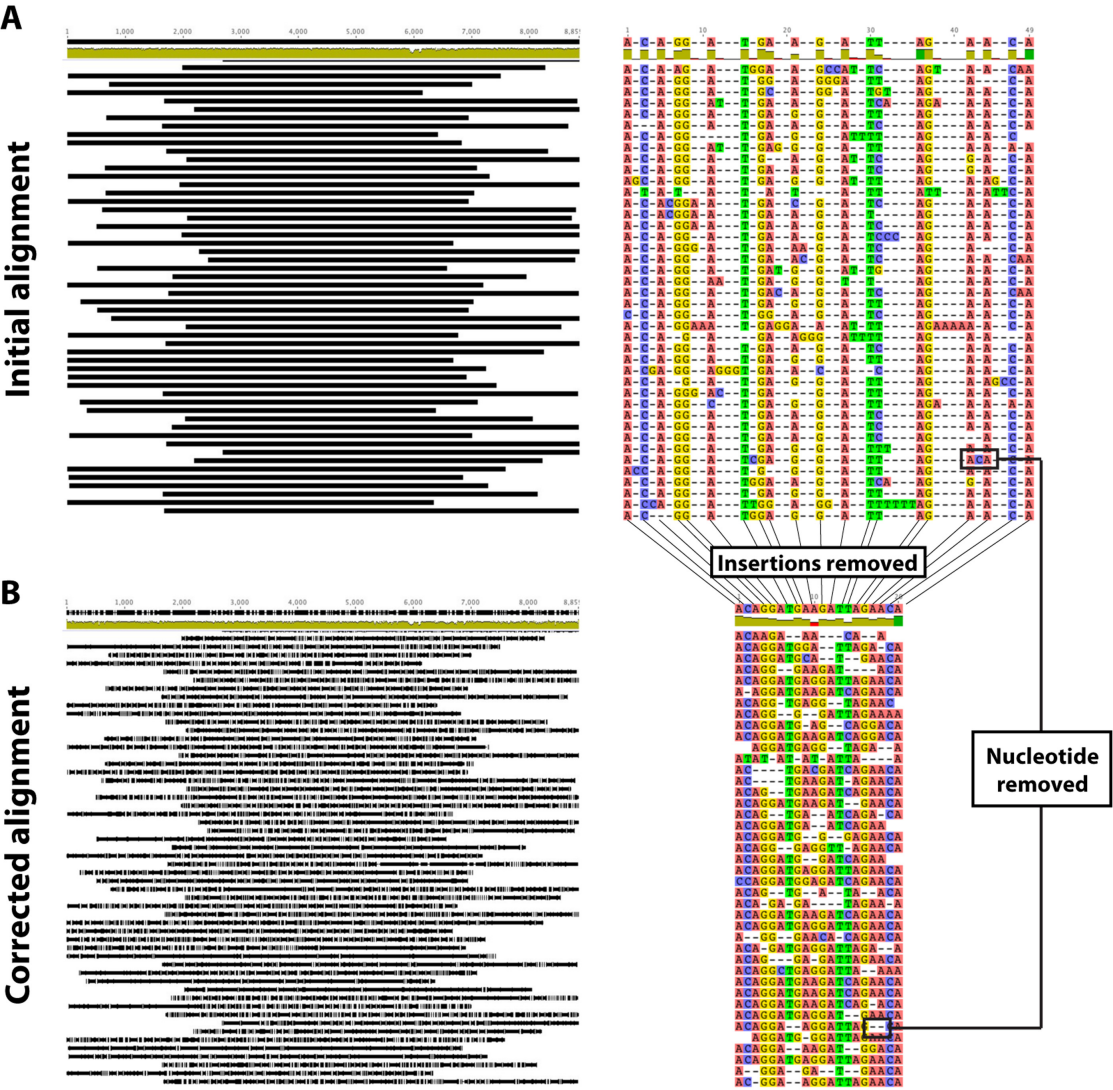
Schematic view of the ‘alignment correction’ procedure implemented during the analytical approach. During implementation of the algorithm, the original alignment (**A**) obtained using Quiver is edited and the positions most likely to lead to errors in the alignment process due to erroneous technique-driven insertions are removed, generating a corrected alignment (**B**) that has lost information but which is enriched in the most reliable segments of the reads.

For each SMRTcell, an initial consensus was estimated using Quiver (41) and a standard HIV-1 reference sequence (HXB2). Despite the relatively large number of sequencing errors present in the raw CLR data, sufficient information remains in the reads to allow alignment using Quiver (Figure 2A). An average of 3256 reads greater than 6 kb were obtained per SMRTcell with a median length of 6892 bases (*p*5 = 6164; *p*95 = 7975) (Supplemental Figure S1A). Alignments showed lower coverage at both ends of the genome since reads can start from either the 5′ or 3′ end and not all of them are long enough to span the entire genome (Supplemental Figure S1B).

Initially, we evaluated whether the genetic information present in the reads, although sufficient to build an overall approximate alignment, could also allow for the differentiation of the unique HIV-1 genomes in each library. However, an attempt to derive an accurate nucleotide sequence for each HIV-1 variant present in the original sample by using a distance method over the aligned reads was unsuccessful, confirming that the number of errors present in the raw reads prevented phylogeny-based reconstruction of the individual HIV-1 genomes.

A computational algorithm was, therefore, developed to remove nucleotide calls most likely to be errors prior to phylogenetic analysis. The concept of this ‘alignment correction’ was based on the fact that insertions and deletions, derived by incorrect base calling, impair the construction of an accurate alignment. The alignment of raw reads to the initial Quiver-derived consensus exhibited a large number of INDELs, which are known to be much more frequent than substitution errors in this sequencing system. Considering that any position located next to a potentially erroneous insertion was more likely to suffer from alignment problems, a computational algorithm was developed to use the information from those insertions to clean the alignment by removing from each read any nucleotide that exhibited an insertion either upstream or downstream of the reference sequence base (Figure 2B). Even though a large number of ‘real’ nucleotides were removed through this approach, the final dataset was then enriched in the nucleotides most likely to be correct. This procedure removed a median of 13.9% (*q*25 = 11.06%, *q*75 = 17.8%) of the positions in each read of the alignment (Supplemental Figure S2). After implementation of the alignment correction, the diversity per position tends to increase (Supplemental Figure S3). This result was related to the fact that, when erroneous insertions were located next to a real nucleotide, the chances of finding a nucleotide identical to the consensus would be higher for that region, and given that the alignment algorithm relied on minimizing the differences between reads, erroneous insertions tended to ‘hide’ the true diversity present among the reads.

### Identification of positions with true diversity

**Figure 3.**
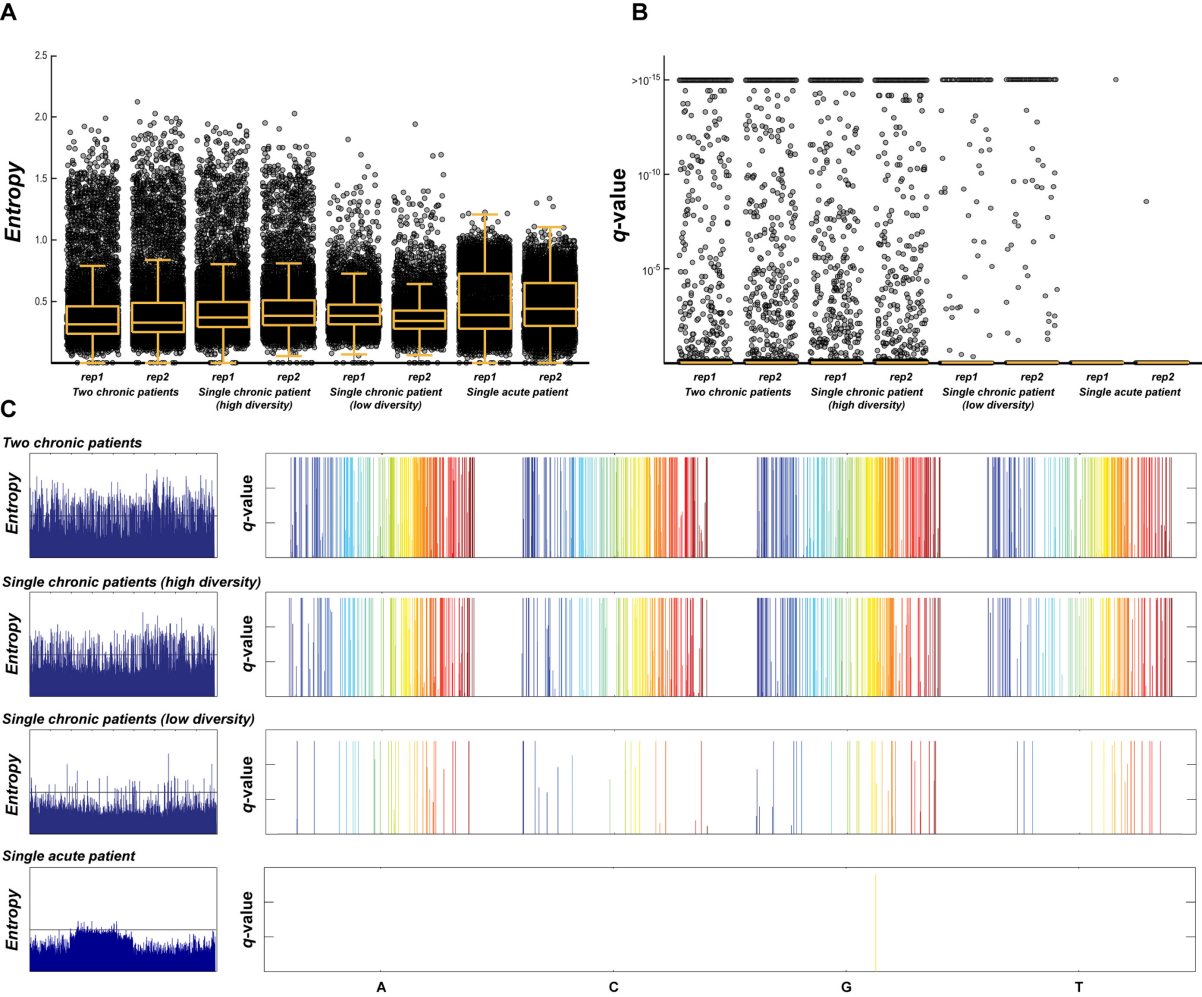
Identification of true diversity among samples with variable degrees of diversity. When the distribution of either the entropy (**A**) or the *q*-values derived from the statistical approach described (**B**) are compared after sequencing a mixture of two HIV-1 chronically infected patients, a single chronically infected patient with high diversity, a single chronically infected patient with low diversity, and an acutely infected patient, the probabilistic approach proves to be more sensitive to variations in real diversity. When both entropy and q-values are used to identify non-consensus nucleotides across the entire HIV-1 genome shown from 5′ (blue) to 3′ (red) (**C**), the differentiation between background noise and real diversity for the statistical approach versus that using entropy is apparent, allowing positions of actual diversity to be identified.

The difficulty in defining true diversity from noise can be seen when examining the variability in observed base frequencies, as measured by entropy, for individual positions in the viral sequence for a mixture of amplicons from acute infection (Figure 3A) or a single SGA (Supplemental Figure S4A). The examined entropy is the simple expectation of negative log probability over the base distribution at individual positions in the multiple sequence alignment. In a situation with no sequencing errors on a clonal sample, the median entropy would be expected to be close to zero. However, average entropies of 0.4181 and 0.4726 were observed in the above datasets, respectively, with 75% of the positions above 0.2911/0.3591. In order to minimize the impact of this overall error, a second algorithm was developed, which weighted positions exhibiting the highest diversity, since these are the positions most likely indicative of true variability. To select those positions, a statistical approach was implemented to analyze only base substitutions, independently of deletions (the alignment-correction algorithm described above having already minimized errors derived from nucleotide insertions). This method assumed that the distribution of these non-consensus nucleotides within the noise followed a binomial distribution with a expected frequency of 0.05, providing a conservative threshold for detecting diversity according to the observed frequency of non-consensus nucleotides when each individual base is analyzed independently from the others (Supplemental Figure S4B) for the acute/single SGA sample. The probability of being a sequencing error was then calculated for each non-consensus nucleotide separately at each position, and corrected for using multiple comparisons by the Benjamini-Hochberg method for defining false discovery rates (FDR) (42). This approach allowed the separation of background noise from true diversity, with the additional benefit of taking into account the number of sequencing reads obtained at each position. Using a *p*-value of 0.05 and *q*-value of 0.2 for this probabilistic approach, the number of positions selected correlated with the diversity in the original sample. In library #1 (mixture of two chronic patients) 930 and 1,010 significant positions were found respectively in each replicate; for library #2 (single chronic patient with high diversity) these numbers were 739 and 614; and for library #3 (single chronic patient with low diversity) 106 and 105 significant positions were found. This is in contrast to library #4 (single acute patient) where a single position in each replicate was identified (*q* < 10^−15^ and *q* = 2.8 × 10^−9^, respectively) (Figure 3B-C). In the acute patient, all the positions except one exhibited a *q*-value of 1, while in the chronic patients 60% of the *q*-values lower than 1 were found to be significant (*q* < 0.2). In addition, 95% of the significant *q*-values were actually lower than 0.01, demonstrating that the probabilistic approach exhibited very low background noise (Figure 3B and C).

### Separation of reads using clustering methods

**Figure 4.**
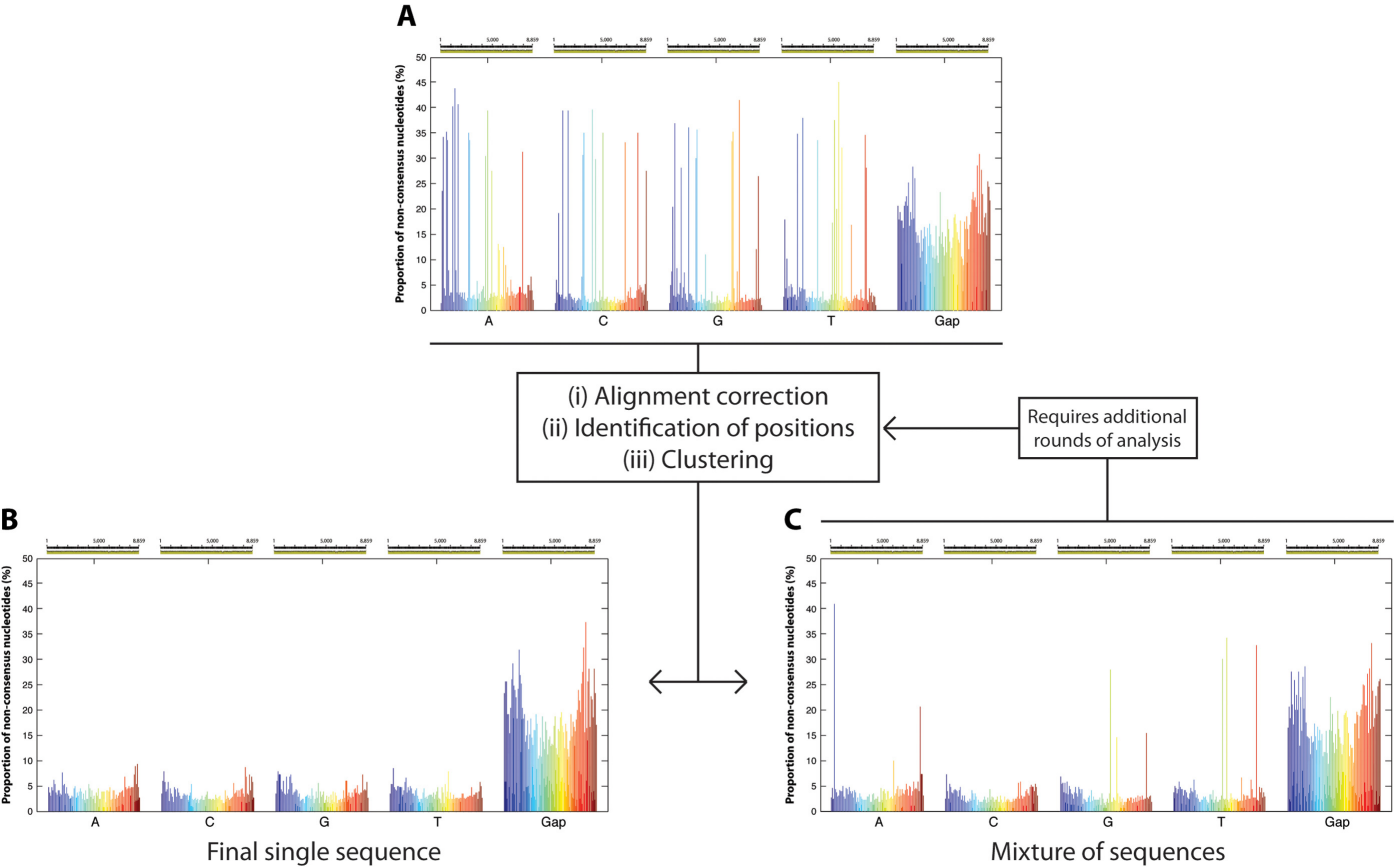
Stepwise classification of reads. Because a majority of the errors generated during sequencing are INDELS, by analyzing the data independently by nucleotide (A, C, G, T), it is possible to reduce the effective background error rate and more accurately identify the positions exhibiting real diversity (**A**). By implementing a clustering analysis only on positions of the alignment exhibiting significant diversity, so that two groups are generated at every step, it is possible to eventually obtain a subgroup of reads that lack any evidence of diversity (**B**). Such homogeneous subgroups can be considered to be the result of sequencing DNA molecules with the exact same nucleotide sequence. Some subgroups require further analysis to derive additional subgroups as they remain a mixture of multiple sequences (**C**).

**Figure 5.**
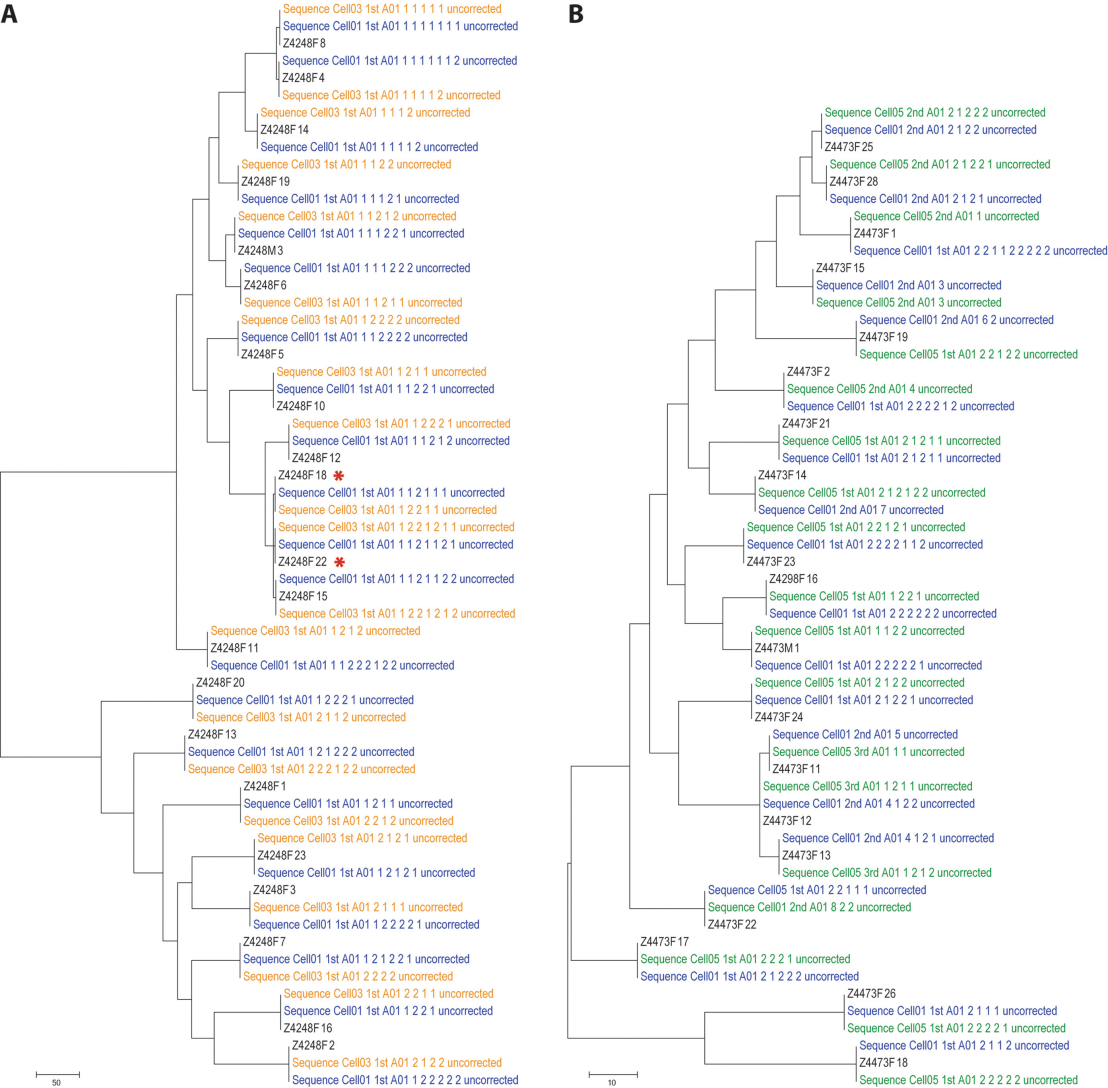
Comparison of the reference Sanger sequences with the consensus sequences generated by the algorithm. Maximum-parsimony trees were constructed using the Sanger reference sequences (labeled in black) for chronic patient Z4248F (**A**) and chronic patient Z4473F (**B**) and the consensus sequences obtained with the algorithms described here (labeled in blue for SMRTCell #1, in orange for SMRT Cell #3 and in green for SMRT Cell #5). The name of each consensus sequence denotes, in addition to the specific SMRT Cell, the round of analysis (first, second or third) and the ‘path’ indicating the subgroup in which the sequence was found. The ‘uncorrected’ label indicates that these are the consensus sequence obtained before implementing the INDEL correction algorithm.

In order to ultimately reconstruct each of the HIV-1 genomes included in the sequencing run, a distance-based clustering method was implemented. This method uses the positions identified above as having significant diversity, to classify the reads into different groups according to their similarity to each other. Instead of using a fixed distance cut-off to separate reads, an analytical approach was employed in which reads were separated sequentially into two groups at a time based on the largest distance between reads determined using the furthest neighbor method (Figure 4). By using this approach it was not necessary to set a specific cutoff and the accuracy of separating different HIV-1 genomes into discrete groups of sequencing reads was increased. Although both groups would likely still have a mixture of reads obtained from different genomes, by sequentially repeating the steps of alignment correction, identification of positions, and clustering, we were able to separate reads into groups of increasing sequence homogeneity. This was repeated until no further positions with significant diversity were found, indicating that the alignment in the final subset was composed exclusively of reads obtained from the sequencing of the same HIV-1 genome (Figure 4A–C). This procedure generated a number of HIV-1 genomes that was equal to, or higher than, the number of HIV-1 genomes present in the original sample. However, phylogenetic analyses showed that this was due to the generation of redundant identical genomes at different cycles of the analysis. In other words, reads belonging to the same genome were at some point in the analysis separated into different subsets and eventually used to independently derive the same HIV-1 genome. Since this diluted the number of reads clustered per genome, and because these redundant genomes were easily identified by phylogenetic analysis, an additional step was included in which all the reads that independently derived the same genome were merged and re-tested by the code to determine whether it constituted a mixture or was derived from the same HIV-1 genome. After this procedure, the number of consensus sequences obtained was equal to the number of SGAs present in the original sample, whether the analysis was started with a mixture of 19 (library #1), 21 (library #2) or 40 defined genomes (library #3) (Figure 5A and B). This result also provided confirmation that each SGA was indeed unique and not a mixture of multiple HIV-1 genomes co-amplified in the same RT-PCR.

### Final correction for single base deletions/insertions

**Figure 6.**
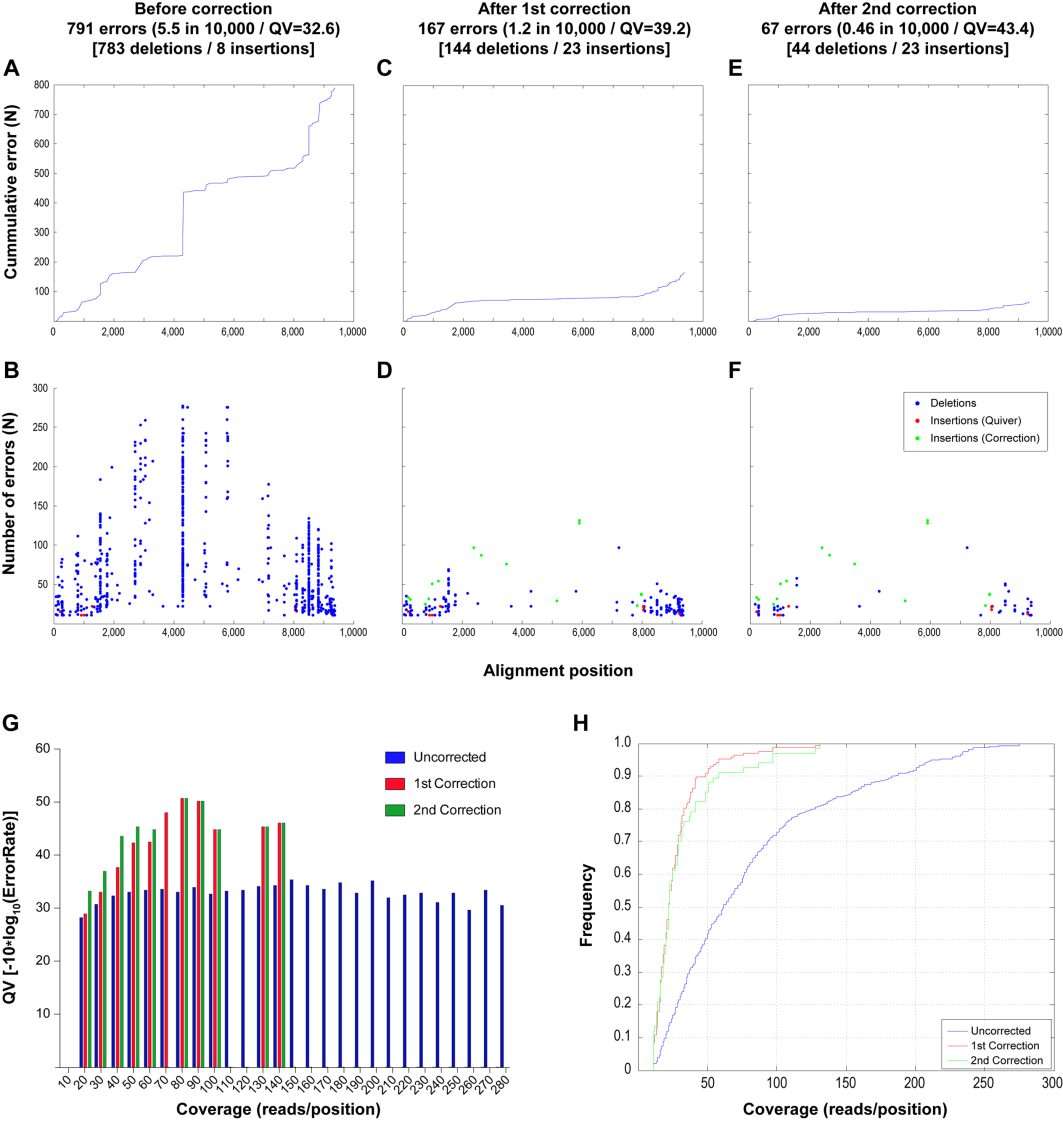
Analysis of errors found before and after implementation of INDEL correction algorithms. The cumulative and absolute number of errors across the alignment is shown for the sequences obtained before performing INDEL correction (**A**, **B**) and after performing the first (**C**, **D**) and second (**E**, **F**) INDEL correction. In addition, the QV according to depth of sequence coverage is shown (**G**) for the uncorrected alignment (blue) as well as for the alignment after the first (red) and second (green) correction. Panel h. shows the cumulative frequency of errors against coverage, showing that before implementation of the correction algorithm, the errors were frequent even at positions with high coverage, while after correction, 90% of the error was limited to positions with coverage less than 50×.

When the sequences obtained using the above algorithm were compared with the sequences obtained by Sanger sequencing, 791 errors (783 deletions and eight insertions) were observed in the total dataset (∼1.6 million nucleotides), which represents a median of 5 (*p*95 = 9, max = 17) errors in each of the 161 genomes analyzed (QV = 32.6). As shown in Figure 6A and B, these errors were most frequent at both ends of the alignments where the sequence coverage was lower. However, clear hotspots of error were identified as shown in Figure 6B as well as several errors in regions with high coverage as shown in Figure 6G (blue bars).

Given that the vast majority of errors were deletions, a probabilistic algorithm (see Materials and Methods, Error Correction Algorithm #1, ECA1) was developed to determine whether an actual position in the alignment was initially removed because of low frequency detection of the nucleotide during sequencing. This algorithm reanalyzes all of the nucleotides initially classified as potentially erroneous insertions in the Quiver alignment for evidence of specific nucleotides present at frequencies significantly higher than that expected for noise. By implementing this new correction algorithm it was possible to reduce the total number of errors from 791 to 167 (144 deletions and 23 insertions, QV = 39.2) with a median of 0 (*p*95 = 4, max = 9) errors in each of the 161 genomes analyzed. The errors that remained were associated with positions of low coverage at the ends of the alignment (Figure 6C and G—red bars), with 90% of the errors located in positions with coverage lower than 50× (Figure 6H). After correction, the majority of hotspots were eliminated (Figure 6D). While the number of deletions was reduced by 80%, the number of insertions increased from 8 to 23. This is due to the fact that given the probabilistic nature of the analysis, a number of false positives were expected, which in this case is 2.3% (15/639), a number close to the 1% cutoff defined by the 0.01 *q*-value.

In order to further reduce the number of errors, an additional approach was explored. The 144 remaining deletions were all single gaps that could be identified by aligning the sequences obtained from the first correction with a reference HIV-1 sequence. To address this, we developed an algorithm that determined, for each of these gaps, whether a nucleotide was present at a significant frequency in at least one of the 50 replicates performed during ECA1 (see Materials and Methods, Error Correction Algorithm #2, ECA2). It is important to note that for this analysis, no information from the known Sanger sequence is required. After implementing the second correction, 100 nucleotides that fit the defined criteria were found. All of them were found to be real nucleotides after comparing with the Sanger reference sequences. In other words, for any given nucleotide initially classified as a technique-driven error that would fill a gap in the alignment, and is identified in at least one of the 50 replicates performed in ECA1, the empiric probability for that nucleotide to be real is 1. After this second correction all 23 insertions remained.

After performing both corrections, the analytical approach developed in the present study was able to derive the sequence for 99.8% of the targeted bases of the 19 different HIV-1 genomes present in library #1 with an error rate of 0.0009% (or 0.9 in 100 000 nucleotides sequenced or QV50); 99.9% of the 21 different HIV-1 genomes present in library #2 with an error rate of 0.003% (or 3 in 100 000 nucleotides sequenced or QV45); and even with 40 different HIV-1 genomes present in library #3, 98.1% of all sequences were obtained with an error rate of 0.007% (or 7 in 100 000 nucleotides sequenced or QV41). In this way we were able to distinguish 9kb genomes differing in only five bases (Figure 5, denoted by asterisks). Additional information on parameters related to the final sequences can be found in Supplementary Figure S5.

### Assessing sensitivity to detect minor variants

In the previous analyses, we had determined the sequences of individual amplicons in mixtures with equal proportions of each. Since direct amplification of a diverse population of viruses would likely involve varying proportions of different variants, we performed SMRT sequencing of a mixture of 20 SGAs from the two chronic patients under study (10 SGAs per patient) in which each one of the SGAs is present at decreasing proportions in the initial sample (Table 1).

**Table 1.** Expected and observed relative frequency of genomic variants during assessment of sensitivity

SGA	Amount in INPUT (ng)	Expected proportion in INPUT (ng loaded/total)	Amount in OUTPUT (number of reads)		Observed proportion in OUTPUT (number reads in SGA/total reads)	
			Replicate 1	Replicate 2	Replicate 1	Replicate 2
*Donor #1*						
Z4248F_1	750.00	25.00	628	526	19.44	19.43
Z4248F_2	375.00	12.50	254	281	7.86	10.38
Z4248F_3	187.50	6.25	177	169	5.48	6.24
Z4248F_6	93.75	3.13	39	34	1.21	1.26
Z4248F_10	46.88	1.56	45	51	1.39	1.88
Z4248F_11	23.44	0.78	ND	ND	ND	ND
Z4248F_13	11.72	0.39	ND	ND	ND	ND
Z4248F_15	5.86	0.20	ND	ND	ND	ND
Z4248F_20	2.93	0.10	ND	ND	ND	ND
Z4248F_23	1.46	0.05	ND	ND	ND	ND
Total reads			1143	1061		
*Donor #2*						
Z4473M_1	750.00	25.00	939	710	29.06	26.23
Z4473F_2	375.00	12.50	541	478	16.74	17.66
Z4473F_12	187.50	6.25	309	269	9.56	9.94
Z4473F_16	93.75	3.13	115	85	3.56	3.14
Z4473F_17	46.88	1.56	42	44	1.30	1.63
Z4473F_19	23.44	0.78	24	30	0.74	1.11
Z4473F_21	11.72	0.39	ND	ND	ND	ND
Z4473F_24	5.86	0.20	ND	ND	ND	ND
Z4473F_25	2.93	0.10	ND	ND	ND	ND
Z4473F_26	1.46	0.05	ND	ND	ND	ND
Total reads			1970	1616		
*Filtered out reads*			118	30	3.65	1.11
Total	3000 ng	100%	3231	2707	100%	100%

ND: not detected.

**Figure 7.**
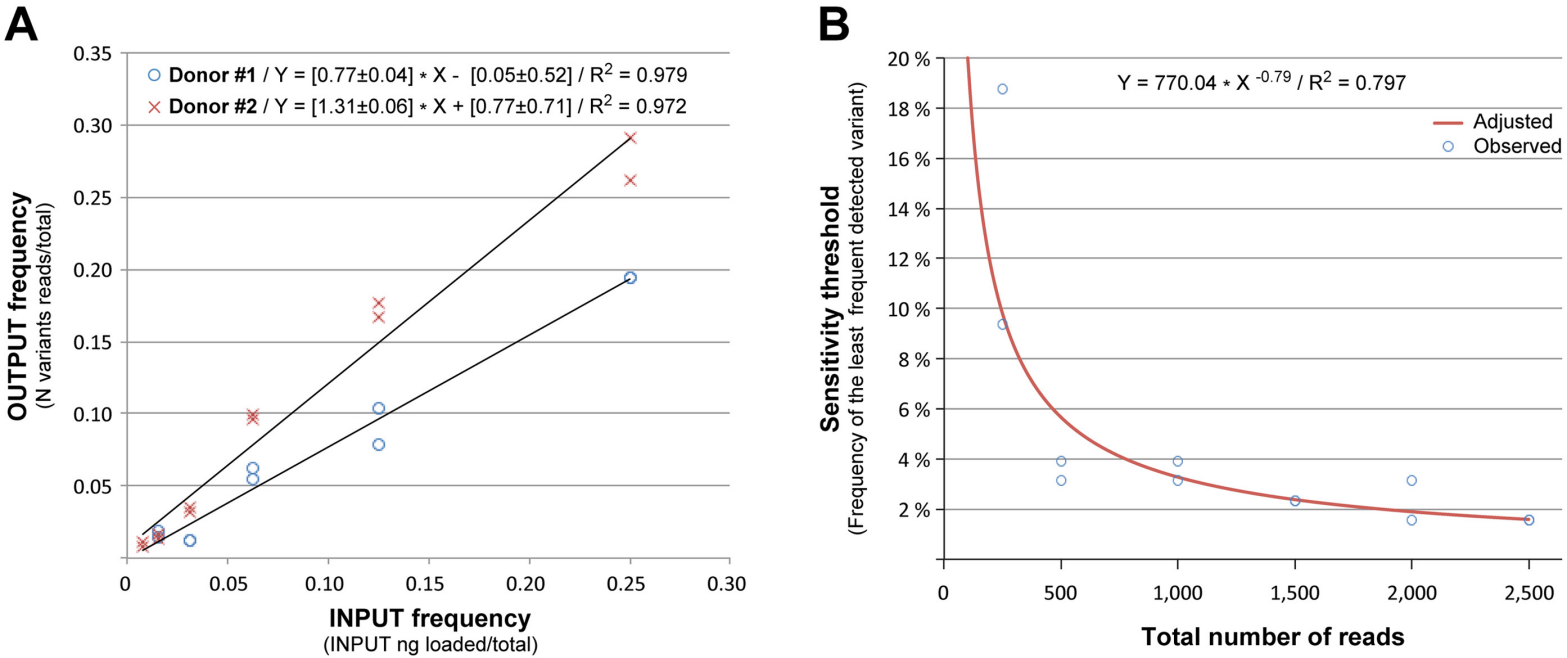
Analysis of sensitivity to detect minor variants. (**A**) A mixture of 20 SGAs from two patients were mixed together at different frequencies and the sequences of each variant was derived using our workflow. As shown there is a strong correlation between the frequency of each variant in the INPUT and the proportion of reads building each variant in the final OUTPUT, indicating that this parameter is a good estimate of frequency in the original sample. (**B**) The same dataset obtained in (A) was resampled for a decreasing number of reads and the sequences of each variant were derived using our workflow. As shown, the sensitivity of our method is strongly correlated in a non-linear manner with the number of reads in the initial INPUT. In particular, every 2-fold increase in the number of reads analyzed leads to a 2-fold increase in the sensitivity to detect minor variants.

The results from SMRT sequencing of this complex mixture of genomes with frequencies ranging from 25% to 0.05%, show that we were able to detect and sequence SGAs present at a frequency as low as 1.56% for patient Z4248F (Donor 1) and 0.78% for patient Z4473F (Donor 2). Moreover, as shown in Figure 7A, there is a highly correlated (*R*^2^ = 0.972; 0.979 respectively) linear relationship between input frequency and the proportion of reads corresponding to each variant indicating that the last parameter is also a measure of frequency in the original sample.

In order to determine how the sensitivity threshold is related to the total number of reads analyzed, we repeated the analysis but after randomly extracting different numbers of reads (250, 500, 1500, 2000, 2500) from the original dataset. As shown in Figure 7B, the sensitivity threshold is dependent on the number of reads generated in the initial sequencing run, such that the sensitivity increases from ∼3.3% with 1000 reads to 1.9% with 2000 reads. This represents a 1.7-fold increase in sensitivity with a 2-fold increase in input read numbers for this dataset. This data is consistent with our ability to detect a minor variant with frequencies ranging from 0.78 to 1.56% from an average of 3000 reads (Table 1). In addition, the analysis of the least frequent variant detected in each of the above data subsets, indicates that a minimum of ∼30 reads >6 kb are required for unambiguous detection (Supplementary Table S1).

Furthermore, when the raw data from both replicates were combined in one single dataset of 5938 reads for analysis, one additional variant per patient was identified (Z4248F_11 and Z4473_21). They average a frequency of 0.58%, consistent with the predicted 0.8% sensitivity for that number of reads in our dataset.

### Development of an automated workflow for sequence analysis

In order to facilitate sequence analysis, the various algorithms for the analytical approaches described above, starting from the raw data (.bax.h5 and .bas.h5 data files) to the final correction algorithm, have been integrated into a single MATLAB^®^ workflow. The final output of this single workflow is a series of FASTA files containing the final corrected sequences.

## DISCUSSION

In the present study, we have developed an analytical workflow that allows for the efficient use of single-pass, continuous long read (CLR) data to interrogate complex mixtures of HIV-1 genomes. We describe a method that can deconvolute mixtures of multiple closely related sequences present at low abundance from raw CLR data. Such deconvolution of full length genomes is not possible with any other available NGS technology.

Importantly, the workflow described does not require the *a priori* definition of the number of putative unique genomes comprising the sample to get an accurate result and explores the entire data set to derive an independent set of unique genetic variants present in the original sample. It is also important to note that the algorithm does not generate any *in silico* artificial sequences or *in silico* recombination of different genetic variants between reads. Based on the results presented here, the correct number and sequence of the different variants present in the original sample can be obtained even when variants differing from each other by as few as five nucleotides are present. Although a limited number of errors remain at the end of the analysis, the final error rate (7/100 000) in the most diverse dataset with 40 different genomic variants at expected frequency of 2.5% is in the order of Sanger sequencing (approx. 1/100 000 to 1/10 000). Given that the error rate is related to sequence coverage, by increasing the number of sequencing reads either by improvements in the sequencing efficiency or simply by running the sample in additional SMRT cells, the error rate can be dramatically reduced. For example, based on sensitivity analyses the workflow is able to detect the least frequent variant with a median coverage of 30×. Current sequencing chemistries (P6-C4) are able to provide around 20,000 reads longer than 6kb. This would yield an expected coverage in the order of 500 reads per sequence, which, would lead to extremely infrequent errors since we have not detected any errors in regions with coverage higher than 150×. Moreover, given that we report a QV41 for samples with a median coverage of 60×, in a 20 000 read dataset there would be the potential to sequence up to 300 HIV-1 genomes at this error rate.

In the present study, we have not sequenced a natural mixture; we artificially built the mixture out of a number of SGAs obtained from patient samples. This was done to allow validation of our workflow, as such validation requires that we know precisely the true underlying DNA sequences of the variants as well as their frequencies in the original sample. There are certain limitations that make it technically difficult to obtain representative full-length HIV-1 genome amplifications from patient samples, such as preferential amplification of sub-genomic mRNAs. As an approach, sensitivity analyses suggest that the workflow is able to derive the sequence of genomic variants present at variable frequencies and up to a detection threshold determined by the number of reads in the input data. Therefore this approach could have direct applicability for natural mixtures, without the need for complementation with lower error-rate, short read NGS technology (e.g. Illumina) to derive the sequence of multiple closely related variants (31). In addition, the fact that the proportion of reads that build each derived nucleotide sequence is strongly correlated with the frequency of the variant in the original sample demonstrates that the workflow can be also used as a quantitative method. Furthermore, because the presented workflow does not rely on CCS reads but only CLR reads, it can readily be implemented on the sequencing of any large amplicons of closely-related genomic variants. Thus, the workflow could have direct applicability to other viral pathogens of similar genomic size, including Influenza viruses, Flaviviruses such as hepatitis C virus or dengue virus, and Parainfluenza viruses such as measles virus or respiratory syncytial virus., For larger genomes the workflow should be able to derive the sequences of each variant for segments of up to 30 kb, or larger genomes by scanning them using a window of 30 kb.

Finally, the methods described here did not require the barcoding of each SGA, but relied exclusively on the information present in the reads to derive each genomic sequence, which were then mapped to each amplicon by their comparison to the Sanger sequences previously obtained. The addition of barcodes to the PCR primers would allow for the possibility of multiplexing multiple samples and relying exclusively on SMRT sequencing for linking the sequences to specific amplicons derived from each sample.

Overall, the results shown in the present study demonstrate that it is possible to overcome the error rate present in raw CLR data derived from SMRT sequencing to obtain highly accurate sequences comprising complex genetic mixtures. This opens the possibility of solving complex sequencing problems that currently lack a solution.

## SUPPLEMENTARY DATA

Supplementary Data are available at NAR Online.

SUPPLEMENTARY DATA
