# Supplementary material for: Multiplexed highly-accurate DNA sequencing of closely-related HIV-1 variants using continuous long reads from single molecule, real-time sequencing
Source: Nucleic Acids Res. 2015 Jun 22;43(20):e129. doi: 10.1093/nar/gkv630 (PMC4787755; doi:10.1093/nar/gkv630)
Supplement: SUPPLEMENTARY DATA [file supp_43_20_e129__index.html]

Multiplexed highly-accurate DNA sequencing of closely-related HIV-1 variants using continuous long reads from single molecule, real-time sequencing — SUPPLEMENTARY DATA 

# Multiplexed highly-accurate DNA sequencing of closely-related HIV-1 variants using continuous long reads from single molecule, real-time sequencing

## SUPPLEMENTARY DATA

- SUPPLEMENTARY DATA
