# Supplementary material for: Multiplexed highly-accurate DNA sequencing of closely-related HIV-1 variants using continuous long reads from single molecule, real-time sequencing
Source: Nucleic Acids Res. 2015 Jun 22;43(20):e129. doi: 10.1093/nar/gkv630 (PMC4787755; doi:10.1093/nar/gkv630)
Supplement: SUPPLEMENTARY DATA [file supp_gkv630_nar-00075-met-k-2015-File009.docx]

**This algorithm can be made available to readers upon request through a Data Transfer Agreement (for academic scientists) or a licence negotiable with the Office of Technology Transfer at Emory University (for commercial entities). All requests should be addressed to Eric Hunter (**[**ehunte4@emory.edu)**](mailto:ehunte4@emory.edu))**.”**

**SUPPLEMENTARY MATERIAL**

**Supplementary text S1. Comparison of probabilistic versus entropy-based approach to identify true diversity:**

Using a p-value of 0.05 and q-value of 0.2 for the probabilistic approach versus a cutoff of 1.0 for the entropy approach, among the 53,441 positions analyzed in the alignments obtained from the six sequencing runs, 3,515 positions (6.6%) were selected by either the entropy criteria or the probabilistic criteria as positions exhibiting variability higher than noise. In both cases, a relationship between the number of positions with high entropy or low q-value and the diversity of the sample in the library was found (Figure 2A-B). However only 2,154 (61.3%) of those 3,515 positions were selected by both approaches (see also Supplementary Figure 5). When we compare the features of the reads selected by either only the entropy criteria or only the probabilistic criteria we found that the sequence coverage differ significantly (p<0.001, Man-Whitney test) between them with a median of 1,430 (q25=881; q75=2,257) reads for the positions with high entropy and non-significant q-values, and a median of 1,838 (q25=1,175; q75=2,346) reads for the positions with low entropy and significant q-values.

**A**


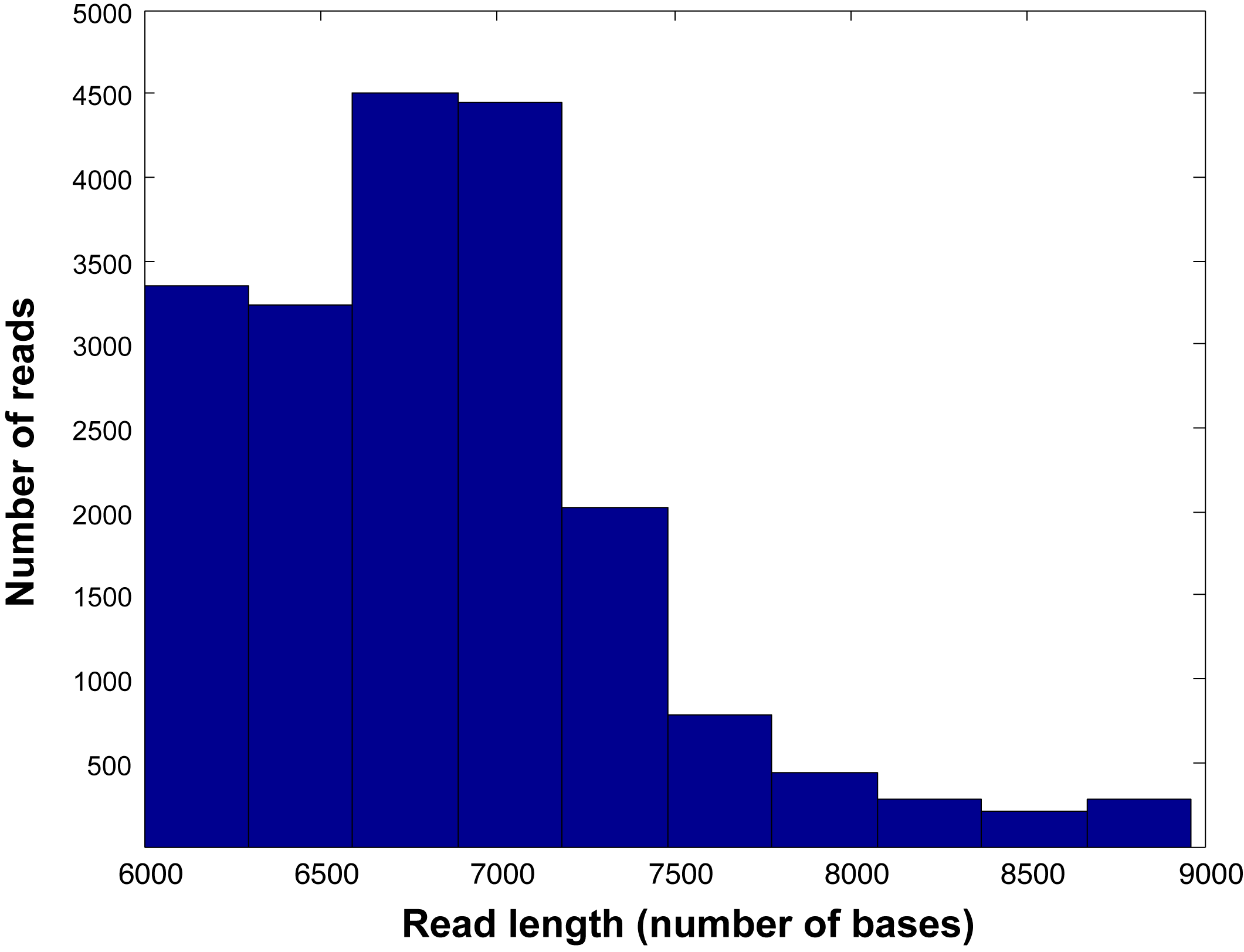


**B**


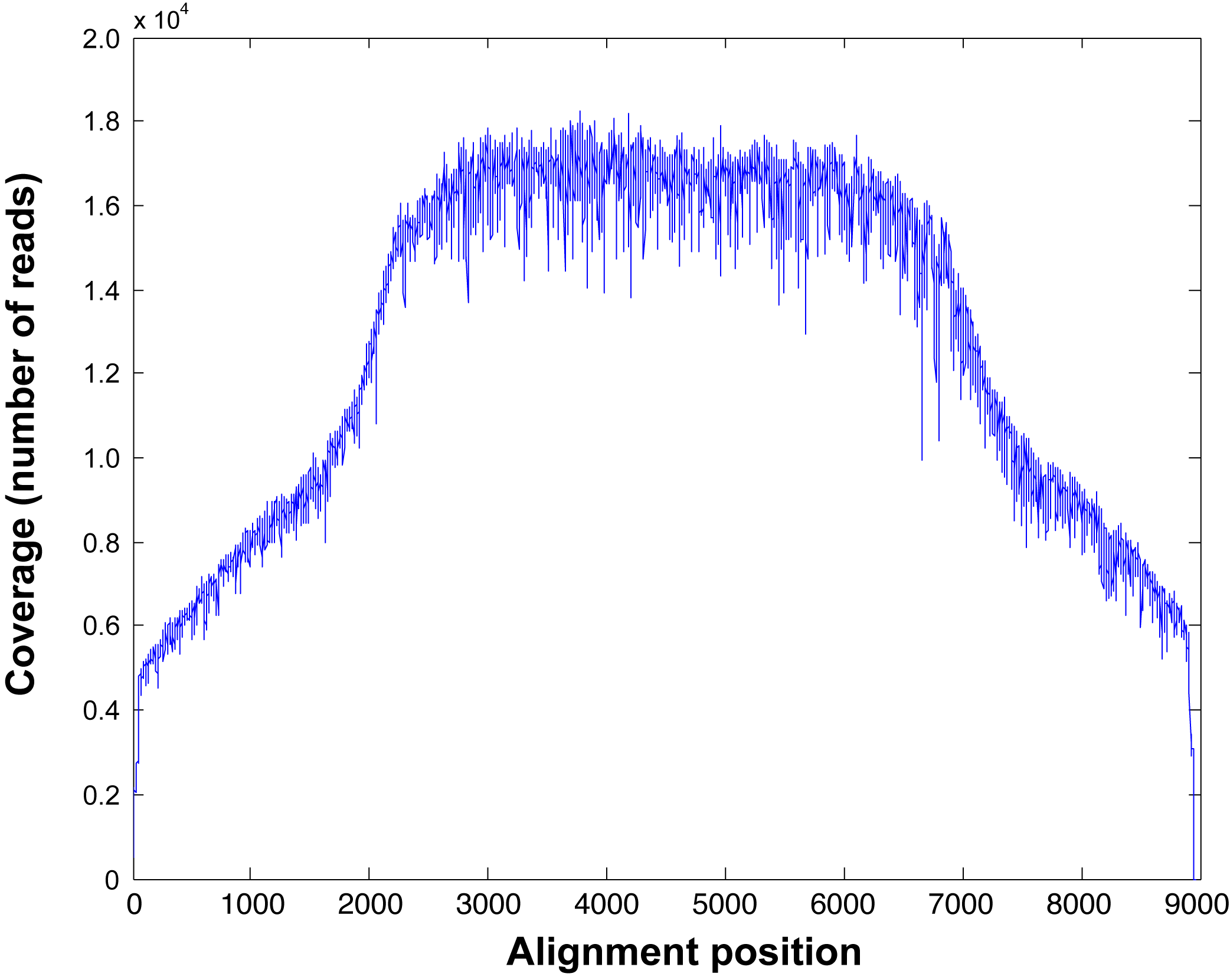


**Supplementary Figure 1. Overall read length and coverage.** In the A panel the distribution of the length of the reads among the 6 cells sequenced is shown. In the B panel the number of sequences (coverage) per position in the alignment is shown.


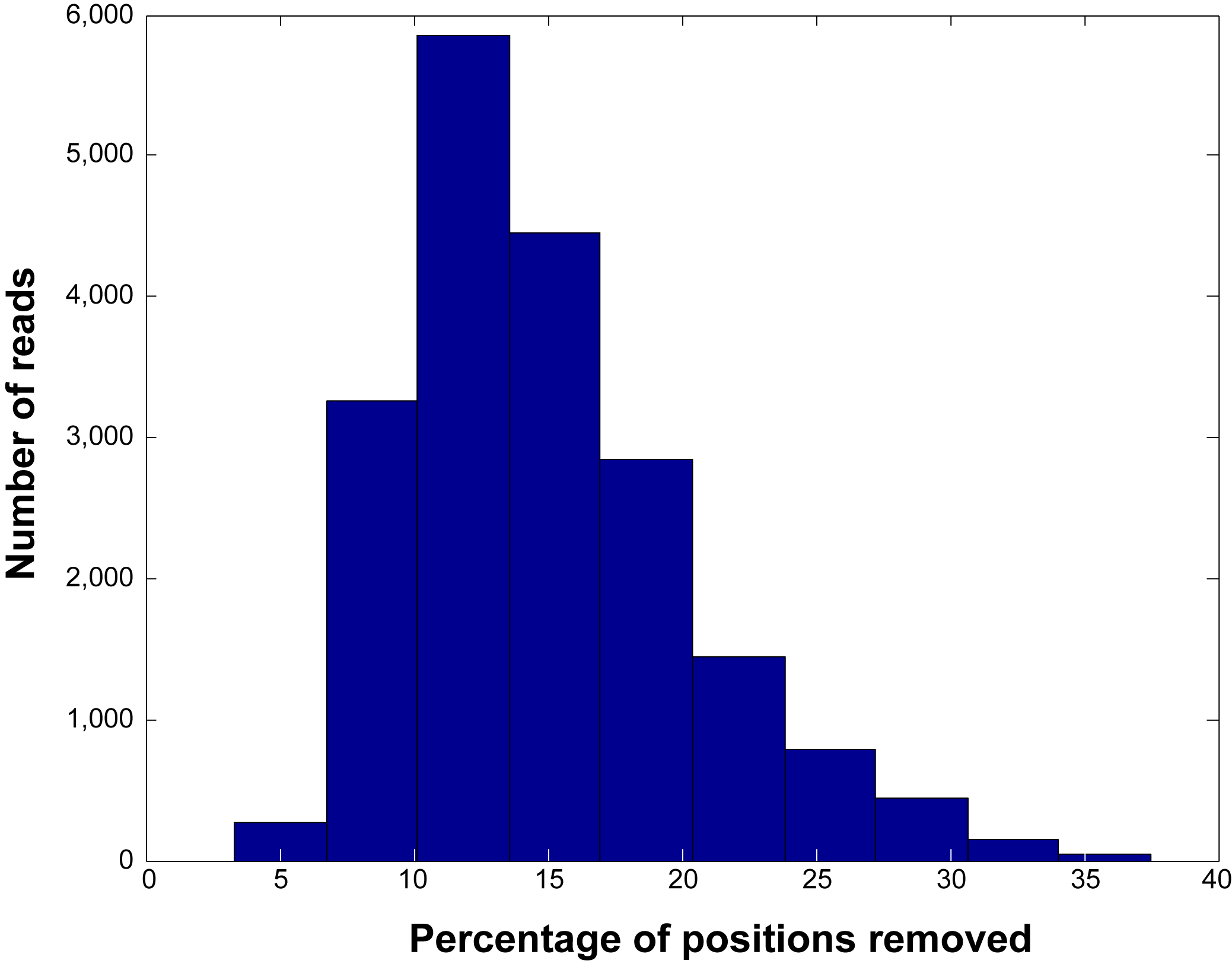


**Supplementary Figure 2. Amount of information removed during implementation of the alignment correction algorithm.** The figure shows the distribution of the percentage of the reads removed during the correction.


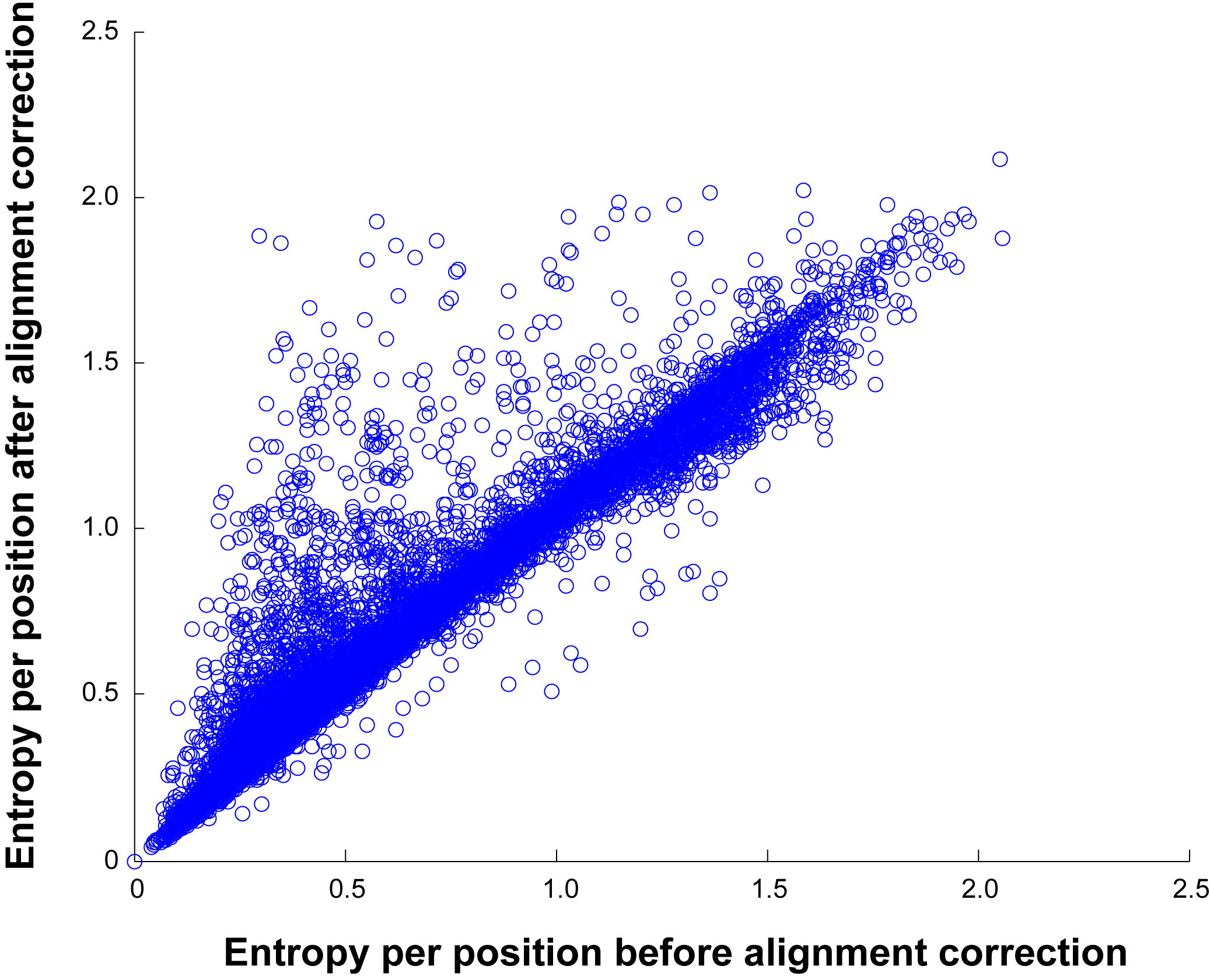


**Supplementary Figure 3. Impact of alignment correction on nucleotide diversity across the alignment.** The figure shows that the correction actually tends to increase the diversity among the positions. This is probably related to the fact that, when erroneous insertions are present among a real nucleotide, the chances to find a nucleotide identical to the consensus are higher and given that the alignment algorithm relies on minimizing the differences between reads, erroneous insertions tend to “hide” the real diversity present among the reads.


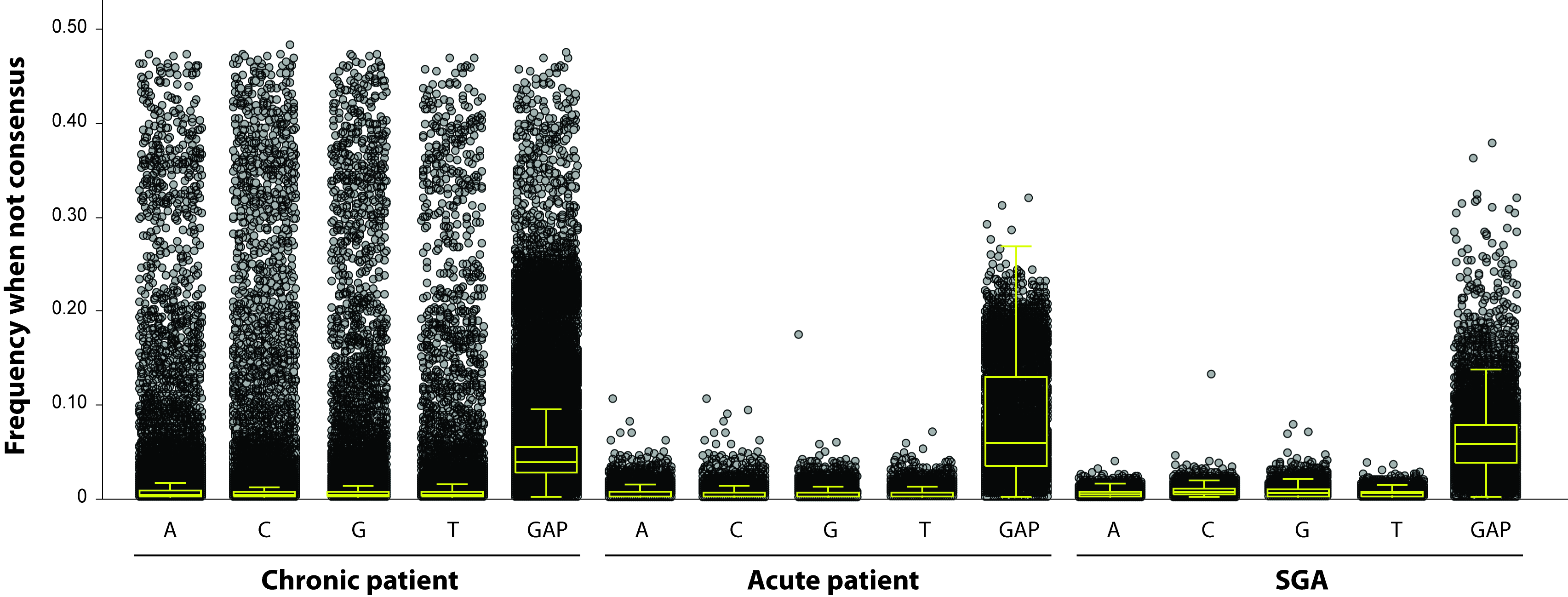


**A**

**B**


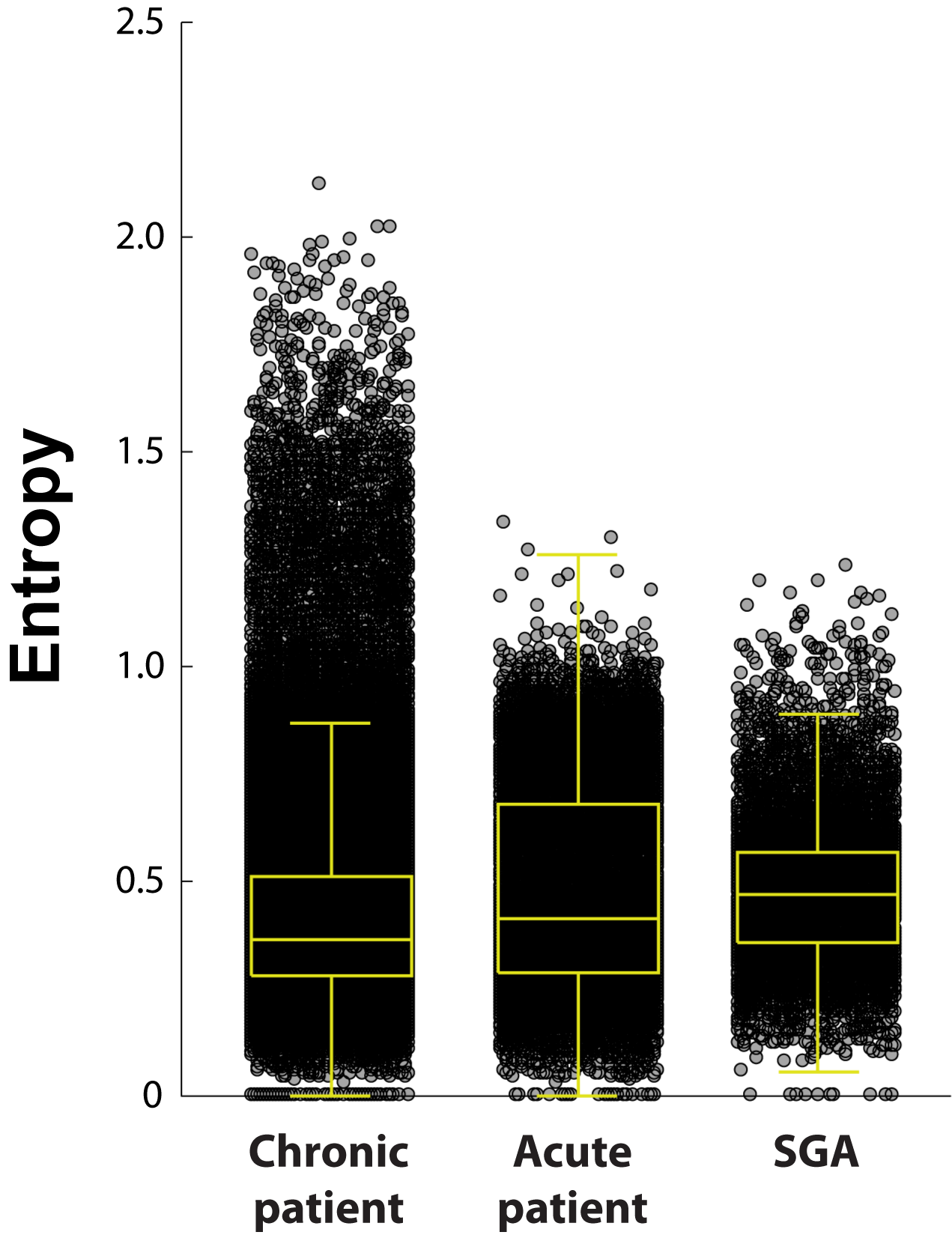


**Supplementary Figure 4. Comparison of diversity between samples exhibiting high and low levels of diversity.** The distribution of the entropy was not significantly different among reads obtained from sequencing a low-diverse acute infection sample or a single SGA from reads obtained from sequencing a high-diverse multi-patients sample (A). The frequency of each of the non-consensus nucleotides separately was clearly different between both settings (B) and suggests that 0.05 sets a maximum expected frequency for the noise. The more clear separation between real diversity and background noise observed in the graph per nucleotide is somehow expected given that the expected frequency of each non-consensus nucleotide (when the nucleotide is an error) is a third of the expected frequency for a mixture of any non-consensus nucleotide excluding gaps. Therefore, the background noise is minimized..

**B**

**A**

**C**

******

******

**D**

******

**E**

**Supplementary Figure 5. Summary of the main output parameters after completing data analysis.** Data is shown as obtained from the Sequencing machine. A01 and A02 are duplicates of library #3, A03 and A04 are duplicates of library #1 and A05 and A06 are duplicates of library #2. The number of reads per final sequence contig was highly dependent on the number of different SGAs present in the original sample (A). The noise, measured as the maximum frequency at which a non-consensus nucleotide was found (B) shows that for most sequences it was lower than 30%. As expected the read length is independent of the genetic diversity of the sample (C). Many genomes were possible to be derived with a coverage as low as 10X (D) while the median coverage was 60x even when 40 different genomes were present in the original sample.

**Supplementary Table 1. Coverage distribution necessary to detect minor variants**

|  |  | **Number of reads in initial dataset** | | | | | | | | | | | |  |
| --- | --- | --- | --- | --- | --- | --- | --- | --- | --- | --- | --- | --- | --- | --- |
|  |  | **250** | | **500** | | **1000** | | **1500** | | **2000** | | **2500** | |  |
| **Precentile** | | ***rep1*** | ***rep2*** | ***rep1*** | ***rep2*** | ***rep1*** | ***rep2*** | ***rep1*** | ***rep2*** | ***rep1*** | ***rep2*** | ***rep1*** | ***rep2*** | **Overall** |
| ***Donor #1*** | |  |  |  |  |  |  |  |  |  |  |  |  |  |
|  | **p5** | 12 | 11 | 10 | 7 | 26 | 5 | 11 | 6 | 8 | 7 | 14 | 12 | **10** |
|  | **p25** | 23 | 17 | 15 | 11 | 37 | 12 | 15 | 10 | 13 | 13 | 17 | 16 | **15** |
|  | **Median** | 36 | 30 | 27 | 19 | 58 | 19 | 25 | 15 | 23 | 20 | 30 | 27 | **26** |
|  | **p75** | 41 | 32 | 28 | 20 | 62 | 20 | 26 | 18 | 23 | 23 | 31 | 32 | **27** |
|  | **p95** | 41 | 32 | 28 | 20 | 62 | 20 | 26 | 18 | 23 | 23 | 31 | 32 | **27** |
| ***Donor #2*** | |  |  |  |  |  |  |  |  |  |  |  |  |  |
|  | **p5** | 10 | 17 | 7 | 4 | 11 | 13 | 10 | 27 | 24 | 24 | 16 | 15 | **14** |
|  | **p25** | 19 | 22 | 10 | 12 | 20 | 19 | 13 | 37 | 38 | 37 | 24 | 20 | **20** |
|  | **Median** | 31 | 37 | 18 | 20 | 33 | 34 | 19 | 62 | 60 | 60 | 36 | 35 | **34** |
|  | **p75** | 33 | 40 | 20 | 21 | 37 | 36 | 21 | 69 | 66 | 67 | 40 | 37 | **37** |
|  | **p95** | 33 | 40 | 20 | 21 | 37 | 36 | 21 | 69 | 66 | 67 | 40 | 37 | **37** |
